# Relationship between BMI and upper gastrointestinal pathologies

**DOI:** 10.1097/MD.0000000000044564

**Published:** 2025-09-19

**Authors:** Przemysław Znamirowski, Łukasz Nawacki, Stanisław Głuszek

**Affiliations:** a Department of General, Oncological and Endocrine Surgery, Regional Hospital in Kielce, Kielce, Poland; b Department of Gastroenterology and Hepatology, K. Gibiński University Clinical Center of the Medical University of Silesia in Katowice, Katowice, Poland; c The Jan Kochanowski University in Kielce, Kielce, Poland; d Holy Cross Cancer Center, Kielce, Poland.

**Keywords:** BMI, gastroscopy, GERD, hiatal hernia, obesity

## Abstract

Obesity is a recognized risk factor for gastrointestinal diseases and is also suspected of aggravating intestinal reflux, causing intestinal atrophy and metaplasia, and an increased risk of carcinogenesis. This study aimed to evaluate the prevalence of upper gastrointestinal pathologies in patients undergoing esophagogastroduodenoscopy procedures, including their relationship to patient body mass index (BMI) values. This retrospective analysis included 368 consecutive outpatients examined by a single physician from March 1, 2023 to February 29, 2024. The analysis was based on the results of endoscopic and histopathological examinations in the patient group. We collected their baseline characteristics. The assessment of endoscopy results included the *Campylobacter*-like organism screening, status of erosive esophagitis, degree of endoscopic signs of gastroesophageal reflux disease as assessed during endoscope withdrawal, status of Z-line displacement as per the Prague classification, degree of Z-line displacement relative to the diaphragmatic branches, and nature of the gastric lake contents. Histopathological assessment focused on the presence of chronic inflammation and the features of active inflammatory processes, foveolar hyperplasia, gastric mucosal atrophy, intestinal metaplasia, and Barrett esophagus. A correlation was observed between the BMI and the prevalence of esophageal hiatal hernia, particularly in relation to large hernias. Interestingly, an inverse relationship was shown regarding the incidence of intestinal reflux. Although biliary reflux and *Helicobacter pylori* infection increased the incidence of intestinal metaplasia in gastric mucosa and Barrett esophagus, these findings were not statistically significant. Furthermore, concomitant thyroid diseases were associated with a higher incidence of biliary reflux and Barrett esophagus. Although BMI correlated with a higher prevalence of large esophageal hiatal hernias, an inverse association was observed with intestinal reflux. Additionally, thyroid diseases were linked to higher incidences of biliary reflux and Barrett esophagus.

## 1. Introduction

### 1.1. Obesity: definition and epidemiology

The World Health Organization (WHO) recognizes the body mass index (BMI, kg/m^2^) as a simple indicator to determine obesity. For adults, a BMI of 25.0 to 29.9 and ≥30 kg/m^2^ is defined as overweight and obesity, respectively. BMI is not used in children and adolescents aged 2 to 18 years; instead, a percentile scale based on the child’s sex and age is recommended.^[1]^ However, in many clinical cases, other parameters, such as the presence of anemia, hypoalbuminemia, and hypoproteinemia, and the number of lymphocytes in the blood smear, should be evaluated to assess a patient’s nutritional status, with BMI values being the least valuable in cancer patients.^[2]^

According to the WHO, 1 in 8 individuals was diagnosed as overweight in 2022. Since 1990, the number of people with obesity has quadrupled. Being overweight affects 43% of adults, whereas 16% are struggling with obesity.^[3]^ Statistical data on the epidemiology of obesity in Poland do not differ from the global trends, with excess body weight being measured in 65.7% of men and 45.9% of women. Approximately 15.4% of men and 15.2% of women have obesity, and 0.5% of men and 0.4% of women have morbid obesity (BMI of ≥40.0 kg/m^2^).^[4]^ In 2022, obesity contributed to the premature deaths of 57,500 Poles, becoming the fourth risk factor for death following hypertension, nicotinism, and metabolic disorders (mainly type 2 diabetes). Notably, the metabolic phenotype of obesity (hyperglycemia, hypertension, and dyslipidemias) is observed in normal-weight patients, possibly due to dietary habits and sedentary lifestyles.^[5]^

### 1.2. Impact of obesity on gastrointestinal function and morbidity

As a pathological condition, obesity affects gastrointestinal morbidity through direct mechanical and physiological effects of excess body fat and indirectly via promoting the state of chronic inflammation and other metabolic effects of excessive body weight. Insulin resistance, increased insulin and leptin levels, and the effects of mediators of chronic inflammation on systemic homeostasis are primary mechanisms underlying morbidity in patients with obesity.^[5,6]^

Excessive body weight contributes to elevated pressure within the gastric cavity, a higher incidence of esophageal hiatal hernias, impaired esophageal motility, and increased transient relaxation and reduced resting tone of the lower esophageal sphincter.^[6,7]^ Together, these factors contribute to a heightened risk of symptomatic gastroesophageal reflux disease (GERD), which is primarily associated with central obesity.^[8]^

Over the past 30 years, the incidence of esophageal adenocarcinoma has significantly increased in Western countries, possibly linked to the parallel increase in the prevalence of obesity. The relative risk of adenocarcinoma increases with the duration and severity of reflux symptoms, as well as with higher BMI. Factors contributing to this trend include the increasing prevalence of GERD, rising obesity rates in Western populations, and the widespread use of medications resulting in the relaxation of the lower esophageal sphincter, thereby facilitating reflux.^[8]^ In contrast, inverse relationships were observed with squamous cell carcinoma, highlighting the need for further investigation to elucidate these findings.^[9]^ A similar relationship was observed between obesity and the incidence of gastric cancer. Additionally, *Helicobacter pylori* infections, a well-recognized risk factor for peptic ulcer disease, also play a significant role in the development of gastric cancer.^[10]^

Among patients undergoing upper gastrointestinal endoscopy screenings, obesity has been considered an independent risk factor for the detection of macroscopic changes in follow-up gastroscopies after previously pathology-free examinations.^[11]^ A positive correlation has been also reported between BMI and the incidence of extraluminal intestinal metaplasia.^[12]^ Obesity was a risk factor for biliary gastropathy in patients who underwent biliary procedures (cholecystectomy and endoscopic retrograde cholangiopancreatography).^[13]^ In histopathological examinations of the specimens collected from patients undergoing revision procedures after laparoscopic sleeve gastrectomy, intestinal reflux was found to induce histological transformation of the gastric mucosa (atrophy, metaplasia). Notably, follow-up studies showed that restoration of the original anatomical relations did not reverse these changes, suggesting the possibility of their perpetuation. This phenomenon appeared independent of *H pylori* infection, as no association was demonstrated.^[14]^ The literature indicates that the presence of bile acids in gastric juice contributes to the progression of mucosal intestinal atrophy and metaplasia without signs of inflammatory infiltrates. In patients with positive *H pylori* infections, this condition further increases the risk of carcinogenesis.^[15]^ The effect of bile acids depends on the pH and the variability of mucosal responses, depending on the gastrointestinal segment.^[16]^ The molecular mechanisms underlying the effects of pH and bile acids on the esophageal and gastric mucosa are not clearly defined. Proposed interactions include an increased reactive oxygen species concentration in the esophageal mucosa,^[17]^ pH-dependent induction of calcium ion-dependent intracellular signaling pathways,^[18,19]^ induction of the expression of cellular receptors (e.g., TGR 5),^[20,21]^ promotion of genomic instability through double-strand DNA breaks (DNA-SB),^[22]^ and overexpression of the mucin family of matrix proteins: MUC1, MUC4, MUC-5AC, with the latter one expressed only in Barrett esophagus cells.^[23–25]^

Previous reports have suggested that the incidence of various pathologies in the upper gastrointestinal tract during endoscopic examinations and later during histopathological evaluations of biopsy specimens may be correlated with being overweight and obesity based on BMI. These changes may affect the risk of upper gastrointestinal diseases, including those with malignant potential. Accordingly, the relationship between overweight and obesity and the prevalence of pathological changes detected through endoscopic and histopathological examinations of the upper gastrointestinal tract was investigated.

### 1.3. Objective

This study aimed to assess the prevalence of pathologies in the upper gastrointestinal tract in esophagogastroduodenoscopy patients, including its relationship to patients’ BMI values. The relationships between the reporting for upper gastrointestinal endoscopic examinations, indications for examinations (symptoms), and comorbidities and BMI values were assessed. Additionally, the relationships between factors other than BMI and the occurrence of pathological lesions within the upper gastrointestinal tract (as described in known studies) were also assessed.

## 2. Methodology

We conducted a retrospective analysis on 368 consecutive patients who presented for outpatient endoscopic examination of the upper gastrointestinal tract, having undergone only routine diagnostic evaluations before the procedure. The same physician examined all patients from March 1, 2023 to February 29, 2024. The patients provided the standard consent for study participation before the examination.

The patients’ clinical data, including anthropometric information and medical history, were collected using a structured questionnaire completed during the initial interview at the time of routine outpatient admission. This form was a standard component of the institution’s clinical protocol and not developed specifically for the study. Although the questionnaire was initially completed based on patient self-report, the attending physician performing the endoscopy reviewed and verified the information against any available medical records. Consequently, the data used for this retrospective analysis reflects both patient-provided information and physician-validated documentation. It is important to note that, due to the retrospective and clinical nature of data collection, some variability and missing entries occurred (particularly in the anthropometric fields) reflecting routine clinical practice rather than study-related omission.

Endoscopic examinations were performed using Olympus Evis Exera II endoscopes, adhering to the current recommendations.^[25]^ After the camera was placed angularly on the tongue, the epiglottis was visualized, and then the camera was moved to the laryngeal part of the pharynx. Esophageal insufflation was performed after swallowing the apparatus to pass the camera through the upper sphincter. The camera was then smoothly inserted into the stomach, and a detailed assessment of the mucosa was postponed until endoscope withdrawal. Once the camera reached the stomach, it was rotated clockwise to visualize the greater curvature. Subsequently, the tip of the endoscope was bent at a right angle to visualize the prepyloric region. Thereafter, it was directed toward the pylorus and then through the duodenal bulb into the descending duodenum. Once the duodenal knee was visualized, the tip of the camera was twisted clockwise to enter the descending part of the duodenum and visualize the region of the major duodenal papilla (papilla of Vater). From this point, the gastrointestinal mucosa was evaluated in detail. In the descending portion of the duodenum, the mucosa, submucosal lesions, and any observable impressions were assessed. In cases where endoscopic procedures were conducted to rule out visceral disease in patients with anemia, biopsy samples were collected from the duodenal bulb and the descending duodenum.

Images were obtained during insertion and withdrawal to ensure a thorough evaluation of the corporeal gastric mucosa. During withdrawal, the endoscope was bent 180° and gently rotated while being withdrawn to carefully assess the mucosa of the gastric angle, lesser curvature, fundus, and subcardial region, along with the impressions originating from diaphragmatic branches. The evaluation focused on mucosal appearance, submucosal changes, and external impressions. Descriptions of pathological changes included evaluation of the degree of related luminal constriction and relation to other gastric landmarks. Specimens for rapid urease tests (*Campylobacter*-like organism [CLO] tests) were routinely taken from the antrum and angle of the stomach. Polyps were evaluated and removed when indicated (including for histopathological verification of the initial diagnosis). All tissue specimens were fixed in 4% buffered formaldehyde for 12 to 24 hours, embedded in paraffin, and sectioned at 4 µm thickness for histological evaluation. Routine hematoxylin and eosin staining was performed. Immunohistochemical staining was conducted using the Ventana ultraView Universal DAB Detection Kit on the Benchmark Ultra platform. Markers such as CK20 and CDX2 were used to identify intestinal metaplasia, while Alcian blue staining was employed for goblet cells.

After ensuring that the endoscope was not twisted, it was withdrawn from the gastric lumen to assess the cardiac function and measure the distance between the gastric cardia and the incisors. Upon withdrawal through the esophagus, detailed reevaluation of the mucosa, submucosal lesions, and impressions, including the location of impressions originating from diaphragmatic branches, were carefully evaluated. The area of the Z-line was carefully examined and, if displaced, described based on to the Prague classification (C: circumferential section, M: maximum length).^[26]^ Specimens for the diagnosis of GERD were collected based on the Seattle guidelines.^[27]^ Erosive esophagitis and candidiasis were evaluated based on the Los Angeles (LA) and Kodsi classifications, respectively.^[28,29]^ Esophageal varices were evaluated based on the Organization Mondiale d'Endoscopie Digestive classification (MST 3.0) with a complementary description per the Baveno classification.^[30]^ The device was slowly withdrawn from the esophagus to minimize the risk of inducing a gag reflex. Whenever feasible, the laryngeal entrance was also evaluated during the withdrawal.

### 2.1. Statistical analysis

The following histopathological findings were considered in the statistical analysis:

1. Chronic inflammation: the presence of inflammatory cells (lymphocytes, plasmacytes, sometimes neutrophils) within the submucosal layer and gastric mucosa.
2. Active inflammation: predominance of neutrophils, erosions, and ulcerations with signs of tissue congestion.
3. Foveolar hyperplasia: excessive growth or proliferation of gastric epithelial cells.
4. Mucosal atrophy: atrophy of the cells lining the stomach.
5. Intestinal metaplasia: presence of goblet and Paneth cells in the gastric mucosa; epithelial type changes.
6. Dysplasia: abnormal organization of the epithelial structure, atypia, including changes in cell shape and size (polymorphism), staining (hyperchromasia) of cell nuclei, and increased mitotic activity; the changes are not accompanied by infiltration extending beyond the local mucosa; low-grade and high-grade dysplasias are being distinguished.
7. Barrett esophagus: epithelial type being changed from squamous multilayered epithelium to cylindrical monolayered epithelium (intestinal metaplasia), the presence of goblet cells (the key histopathological marker).

Additionally, the stomach was assessed for the presence and nature of polypoid lesions.

The results were subjected to statistical analysis. Data were collected in a Microsoft Excel spreadsheet (Microsoft Corporation, Redmond). Statistical analyses were conducted using Statistical for Windows ver. 13.1 (TIBCO Software Inc., StatSoft, Poland). Each of the evaluated endoscopic and histopathological findings was tested for correlations with quantitative and qualitative variables using appropriate logistic regression models. Logistic regression analysis facilitated the identification of predictors affecting endoscopic and histopathological findings. The final form of the logistic regression model with qualitative and quantitative explanatory variables was used to identify the determinants affecting this phenomenon. Model fit was evaluated using the Hosmer–Lemeshow test. To ensure appropriate model result interpretation, *P* > .05 was set for the Hosmer–Lemeshow test. The chi-squared test of independence was applied to analyze relationships between nominal variables or between nominal and ordinal variables. Statistical significance was determined at *P* ≥ .05. The Shapiro–Wilk test was used to verify the normality of the distribution of BMI values as a function of the aforementioned factors. The alternative hypothesis that the examined trait does not follow normal distribution was adopted for *P* < .05. If the examined variables had a non-normal distribution, the results were expressed as medians and the first and third quartiles (Q1 and Q3). Pearson correlation was used to assess the impact of independent variables on the chances of particular events. Nonparametric Mann–Whitney *U*-test was used to compare individual quantitative variables between groups of independent samples, whereas nonparametric Kruskal–Wallis test was used to compare individual quantitative variables between more than 2 groups of independent samples. Missing data were excluded from the analysis of the relationships between BMI and the results of endoscopic and histopathological examinations, and the results did not show significant importance.

The study was approved by the decision of The Bioethics Committee of the Medical College of the Jan Kochanowski University in Kielce approved this study (date: July 17, 2024).

## 3. Results

We enrolled 368 patients, with the 5th and 7th decades of life accounting for the largest age groups. Two-thirds of all patients were female, with older groups having female predominance. Tables 1 and 2 show the study group characteristics. Anthropometric data were incomplete in 26 subjects due to changes in the scope of records kept at the institution during the study period and the patients’ failure to complete these data.

**Table 1:** Study participants by age category and sex.

	Women, n (%)	Men, n (%)	Total, n (%)
Age categories (years)			
** **18–30	19 (4.9%)	16 (4.3%)	35 (9.5%)
** **31–40	35 (9.5%)	20 (5.5%)	55 (14.9%)
** **41–50	49 (13.3%)	33 (9.0%)	82 (22.3%)
** **51–60	38 (10.3%)	25 (6.8%)	63 (17.1%)
** **61–70	56 (15.2%)	28 (7.6%)	84 (22.8%)
** **>70	33 (9.0%)	16 (4.3%)	49 (13.3%)
** **Total	230 (62.5%)	138 (37.5%)	368 (100%)

**Table 2:** Basic statistics of BMI in the study group.

	*M*	Me	Q1	Q3	SD	Minimum	Maximum
Weight (kg)	75.56	73.00	64	85	17.12	40	150
Women	69.89	69.5	60.25	75.75	14.54	40	140
Men	85.0	84.0	73.8	93.0	16.9	47	150
Height (cm)	168.14	168.0	161.0	175.0	8.82	149	193
Women	163.54	164.0	160.0	168.0	5.89	149	186
Men	175.8	176.0	170.0	181.0	7.5	150	193
BMI (kg/m^2^)	26.64	26.22	23.05	29.07	5.26	16.65	54.69
Women	26.15	25.68	22.70	28.84	5.36	16.96	54.69
Men	27.5	26.90	23.9	29.4	5.0	16.7	44.30

BMI = body mass index, *M* = mean, Me = median, Q1 = 1st quartile, Q3 = 3rd quartile, SD = standard deviation.

The most common indications for endoscopy included dyspepsia (198 patients, 54.7%), and GERD (134 patients, 37.02%), including heartburn (73 patients, 20.17%), and other GERD symptoms (61 patients, 16.85%). This group consisted of patients with chronic laryngitis referred by ENT specialists for endoscopic evaluation of possible GERD symptoms.

We collected the patients’ medical history using a structured questionnaire filled out and completed during the interview before study inclusion. The interview included questions regarding anthropometric parameters, such as age, body weight, and height. Comorbidities included hypertension (83 subjects, 22.93%), diabetes mellitus (no breakdown by type: 30 subjects, 8.29%), ischemic heart disease (8 subjects, 2.21%), history of myocardial infarction (3 subjects, 0.83%), cardiac arrhythmia (6 subjects, 1.66%), history of stroke or transient ischemic attack (3 subjects, 0.83%), thyroid disease (36 subjects, 9.94%), liver disease (11 subjects, 3.04%), bronchial asthma (9 subjects, 2.49%), kidney failure (5 subjects, 1.38%), musculoskeletal disease (arthritis, spinal diseases, back pain: 35 subjects, 9.67%), and mental disorders (13 subjects, 3.59%). Regarding the use of stimulants, only 40 patients (11.05%) in the study group admitted to nicotinism.

Regarding medical and surgical history, 8 (2.17%) patients underwent oncological treatment (5 cases of breast cancer, 1 case of lung cancer, 1 case of esophageal cancer, 1 case of colon cancer), and 134 (36.41%) underwent surgery. The most common procedures in this group included cholecystectomy (30 subjects, 8.29%), appendectomy (33 subjects, 9.12%), cesarean section (29 subjects, 8.01%), and other gynecological surgeries (26 subjects, 7.18%).

In the evaluation of the esophagogastric junction, 77 patients (20.92%) had erosive esophagitis, of which, 37 (10.0%), 24 (6.5%), 13 (3.5%), and 3 patients (0.8%) had grades A, B, C, and D erosive esophagitis, respectively. Additionally, 22 patients (5.9%) had other flat mucosal lesions near the gastric cardia. Based on the Prague classification, Z-line displacement showed circular section (C) in 16 patients (4.3%) and long tongues (M) in 61 patients (16.5%), with all displacements being short in length. Furthermore, 73 patients (19.8%) had displaced diaphragmatic branch impressions. Displacements that might have corresponded to a sliding hiatal hernia (≥2 cm) were diagnosed upon endoscopic evaluation in 60 patients (16.3%).

In the evaluation of the gastric body, 58 patients (15.8%) had bile lake. Other commonly observed pathologies in the gastric body included flat areas of altered mucosa (i.e., prominent against the surrounding tissue) (22 patients, 6.0%), papular gastropathy (41 patients, 11.1%), and polyps (18 patients, 4.9%). In the evaluation of the antrum, erythematous/papular gastropathy (264 patients, 71.7%) and erosive gastropathy (38 patients, 10.3%) were the most common. Four patients (0.1%) had active peptic ulcer disease.

Upon endoscope withdrawal, 105 patients (28.5%) had gastric cardia insufficiency, including 57 with insufficiency over a segment of ≥2 cm (15.5%). Other findings from the endoscopic examination were omitted from the statistical analysis (but not from the examination) due to their sporadic prevalence.

During the examination, biopsy specimens were collected from 313 subjects (85.0%), including biopsy specimens taken from 1, 2, 3, or more sites in 163 (44.3%), 121 (32.9%), and 29 (7.8%), respectively. Statistically, a mean of 1.33 biopsies were obtained per examination. Analyzing the locations of biopsies, the specimens were most frequently collected from the antrum (278 examinations, 56.6% of biopsies), cardia (81 examinations, 18.7% of biopsies), and gastric body (80 examinations, 18.5% of biopsies). Additionally, rapid urease (CLO test) screening for *H pylori* infection yielded positive results in 115 cases, accounting for 31.2% of all tests performed.

The relationship between BMI and the extent of esophageal leak in centimeters showed a statistically significant difference (*P* = .042). The highest median BMI was found in the group with leaks measuring ≥4 cm upon endoscope withdrawal. This finding confirms that abdominal obesity is a risk factor for sliding esophageal hiatal hernia in the studied population. Details of these associations are presented in Table 3.

**Table 3:** Assessment of the effect of subjects’ BMI on the prevalence of endoscopic lesions.

	BMI class according to WHO						BMI			*P*, *Z* or *H*
	Underweight	Standard	Overweight	Obese			Me	Q1	Q3	
				Class I	Class II	Class III				
CLO screening										
Negative	7 (1.9%)	86 (23.4%)	98 (26.6%)	29 (7.9%)	9 (2.4%)	5 (1.4%)	26.23	23.03	29.09	*P* = .838 *Z* = −0.203
Positive	2 (0.5%)	41 (11.1%)	43 (11.7%)	13 (3.5%)	5 (1.4%)	3 (0.8 %)	26.15	23.37	29.06	
Esophageal erosions										
LA A	0	9 (2.4%)	18 (4.9%)	1 (0.3%)	2 (0.5%)	0	27.78	25.01	29.06	*P* = .785 *H* = 1.066
LA B	0	6 (1.6%)	6 (1.6%)	12 (3.3%)	2 (0.5%)	0	26.64	24.44	29.61	
LA C + D	1 (0.3%)	6 (1.6%)	1 (0.3%)	5 (1.4%)	1 (0.3%)	1 (0.3%)	28.34	23.76	31.56	
Leaks (withdrawal)										
0	**9 (2.4%**)	**102 (27.7%**)	**91 (24.7%**)	**30 (8.1%**)	**8 (2.2%**)	**6 (1.6%**)	**25.68**	**22.58**	**28.90**	***P*** = **.042** ***H*** = **17.13**
** **1–3 cm	**0**	**25 (6.8%**)	**47 (12.8%**)	**10 (2.7%**)	**6 (1.6%**)	**2 (0.5%**)	**27.50**	**24.95**	**29.74**	
≥4 cm	**0**	**0**	**0**	**3 (0.8%**)	**2 (0.5%**)	**0**	**30.56**	**27.83**	**32.04**	
Prague criterion C (cm)										
Absent	9 (2.4%)	122 (33.1%)	133 (36.1%)	41 (11.1%)	14 (3.8%)	8 (2.2%)	25.21	22.98	28.90	*P* = .527 *H* = −2.224
1–2 cm	0	3 (0.8%)	7 (1.9%)	0	0	0	26.64	24.83	28.76	
3–9 cm	0	0	1 (0.3%)	1 (0.3%)	0	0	28.75	26.29	31.22	
≥10 cm	0	2 (0.5%)	0	0	0	0	23.32	22.83	23.80	
Prague criterion M (cm)										
Absent	9 (2.4%)	104 (28.3%)	116 (31.5%)	36 (9.8%)	13 (3.5%)	8 (2.2%)	26.22	23.14	29.09	*P* = .508 *H* = 3.304
1–2 cm	0	17 (4.6%)	13 (3.5%)	3 (0.8%)	0	0	24.14	22.46	28.39	
≥3 cm	0	6 (1.6%)	12 (3.3%)	3 (0.8%)	5 (1.4%)	0	27.61	25.56	29.95	
Impressions (cm)										
Absent	9 (2.4%)	117 (31.8%)	101 (27.4%)	31 (8m4%)	8 (2.2%)	8 (2.2%)	25.61	22.58	28.90	*P* = .605 *H* = 4.507
1–2 cm	0	8 (2.2%)	19 (5.2%)	6 (1.6%)	3 (0.8%)	0	27.78	25.59	29.68	
≥3 cm	0	2 (0.5%)	21 (5.7%)	5 (1.4%)	4 (1.1%)	0	27.64	24.86	29.78	
Bile lake										
Present	**3 (0.8%**)	**28 (7.6%**)	**12 (3.3%**)	**7 (1.9%**)	**3 (0.8%**)	**1 (0.3%**)	**24.06**	**21.96**	**28.04**	***P* = .046** ***Z*** = **1.996**
Absent	**6 (1.6%**)	**99 (26.9%**)	**129 (35.0%**)	**35 (9.5%**)	**11 (3.0%**)	**7 (1.9%**)	**26.36**	**23.45**	**29.28**	
Stagnant gastric lake										
Present	1 (0.3%)	1 (0.3%)	1 (0.3%)	0	0	0	23.66	17.72	27.44	*P* = .238 *Z* = 1.179
Absent	8 (2.2%)	126 (34.2%)	140 (38.0%)	42 (11.4%)	14 (3.8%)	8 (2.2%)	26.22	23.04	29.09	
Food matter in gastric lake contents										
Absent	9 (2.4%)	126 (34.2%)	139 (37.8%)	41 (11.1%)	14 (3.8%)	8 (2.2%)	26.21	23.04	29.06	*P* = .824 *Z* = −0.2219
Present	0	1 (0.3%)	2 (0.5%)	1 (0.3%)	0	0	27.52	22.73	30.04	
Corporeal gastric ulceration										
Absent	9 (2.4%)	126 (34.2%)	139 (37.8%)	41 (11.1%)	14 (3.8%)	8 (2.2%)	26.21	23.04	29.06	*P* = .479 *Z* = −0.706
Present	0	1 (0.3%)	2 (0.5%)	1 (0.3%)	0	0	28.75	23.49	31.14	
Corporeal gastropathy										
Absent	9 (2.4%)	111 (30.2%)	129 (35.5%)	38 (10.3%)	13 (3.5%)	5 (1.4%)	26.21	23.04	29.06	*P* = .511 *Z* = −0.656
Present	0	16 (4.3%)	12 (3.3%)	4 (1.1%)	1 (0.3%)	3 (0.8%)	26.99	23.30	29.39	
Corporeal gastric polyps										
Absent	9 (2.4%)	122 (33.2%)	131 (35.6%)	42 (11.4%)	13 (3.5%)	8 (2.2%)	26.21	22.98	29.29	*P* = .874 *Z* = −0.158
Present	0	5 (1.4%)	10 (2.7%)	0	1 (0.3%)	0	26.67	23.64	28.44	
Corporeal gastric flat lesions										
Absent	9 (2.4%)	119 (32.3%)	135 (36.7%)	37 (10.0%)	14 (3.8%)	12 (3.3%)	26.19	23.09	29.06	*P* = .452 *Z* = −0.750
Present	0	8 (2.2%)	8 (2.2%)	5 (1.4%)	2 (0.5%)	0	26.95	22.98	31.91	
Antral erosions										
Absent	9 (2.4%)	110 (29.9%)	127 (34.5%)	37 (10.0%)	14 (3.8%)	6 (1.6%)	26.68	23.03	29.28	*P* = .592 *Z* = 0.535
Present	0	17 (4.6%)	14 (3.8%)	5 (1.4%)	0	2 (0.5%)	26.24	23.45	28.90	
Antral gastropathy (erythematous and papular)										
Absent	3 (0.8%)	38 (10.3%)	38 (10.3%)	13 (3.5%)	2 (0.5%)	6 (1.6%)	25.71	22.75	29.06	*P* = .992 *Z* = −0.002
Present	6 (1.6%)	89 (24.2%)	103 (28.0%)	29 (7.9%)	12 (3.3%)	2 (0.5%)	26.29	23.42	29.09	
Antral ulceration										
Absent	9 (2.4%)	125 (34.0%)	141 (38.3%)	41 (11.1%)	14 (3.8%)	7 (1.9%)	26.29	23.14	29.06	*P* = .683 *Z* = −0.407
Present	0	2 (0.5%)	1 (0.3%)	1 (0.3%)	0	2 (0.5%)	25.69	20.46	42.49	

BMI data missing: n = 27 (7.3%). Statistically significant values are indicated in bold.

BMI = body mass index, CLO = *Campylobacter*-like organism, *H* = Kruskal–Wallis test, LA = Los Angeles, Me = median, Q1 = 1st quartile, Q3 = 3rd quartile, WHO = World Health Organization, *Z* = Mann–Whitney *U*-test.

Additionally, a statistically significant relationship was also observed between the BMI and presence of bile lakes. Individuals with bile lakes had a significantly lower median BMI (Me = 24.06, *P* = .046), indicating that obesity is not the primary factor for the intestinal contents being backed up to the stomach. Further analysis of bile lake occurrence showed no differences in risk based on sex or age. However, after standardization for comorbidities and history of surgical procedures, thyroid disease and history of cholecystectomy were identified as significant risk factors for bile lakes. History of cholecystectomy increases the risk of bile lake by 83% (OR [odds rato] = 3.989; 95% confidence interval [CI]: 1.932–6.958), whereas in the case of thyroid disease, the risk is increased by 17.53% (OR = 3.826, 95% CI: 1.671–8.115). In this context, a history of thyroid diseases also increased the frequency of the histopathological diagnosis of Barrett esophagus in the study group. Another medical history-related risk factor is history of cancer treatment, increasing the risk of Barrett esophagus by 44.73% (OR = 17.803, 95% CI: 6.309–59.412) and thyroid disease by 35.57% (OR = 3.119, 95% CI: 1.204–8.005). In contrast, no statistically significant relationships were identified between BMI and other specified lesions as observed in the endoscopic assessment.

The analysis of the histopathological data showed no statistically significant correlations between the results and histopathological nature of the lesions during the examination. Consistent with existing literature, the BMI did not influence the prevalence of Barrett esophagus in the study group. Notably, in patients diagnosed with mucosal atrophy and high inflammatory activity, the median BMI was markedly higher than the overall median value, albeit not statistically significant. A detailed analysis of the histopathological traits by BMI category is shown in Table 4.

**Table 4:** Histopathological findings with trait severity scores and statistical assessment of correlation with BMI.

	Total	BMI class according to WHO						BMI			*P*-value
		Underweight	Standard	Overweight	Obese			Me	Q1	Q3	
					Class I	Class II	Class III				
Chronic inflammation	**281 (76.4%**)	**5 (1.4%**)	**88 (23.9%**)	**117 (31.8%**)	**34 (9.2%**)	**13 (3.5%**)	**6 (1.6%**)	**26.45**	**23.72**	**29.35**	*P* = .726 *H* = 6.977
Mild (+)	194 (52.7%)	3 (0.8%)	64 (17.4%)	82 (22.3%)	22 (6.0%)	9 (2.4%)	2 (0.5%)	26.39	23.42	29.28	
Moderate (++)	83 (22.5%)	2 (0.5%)	23 (6.2%)	33 (9.0%)	12 (3.3%)	3 (0.8%)	4 (1.1%)	26.64	26.64	29.68	
Severe (+++)	4 (1.1%)	0	1 (0.3%)	2 (0.5%)	0	1 (0.3%)	0	24.11	21.04	29.42	
Active inflammation	**64 (17.4%**)	**0**	**21 (5.7%**)	**23 (6.2%**)	**8 (2.2%**)	**2 (0.5%**)	**4 (1.1%**)	**26.56**	**23.88**	**29.57**	*P* = .202 *H* = 4.617
Mild (+)	47 (12.8%)	0	19 (5.2%)	15 (4.1%)	5 (1.4%)	1 (0.3%)	3 (0.8%)	26.07	23.62	28.84	
Moderate (++)	15 (4.1%)	0	2 (0.5%)	7 (1.9%)	2 (0.5%)	1 (0.3%)	1 (0.3%)	27.45	26.64	30.47	
Severe (+++)	2 (0.5%)	0	0	1 (0.3%)	1 (0.3%)	0	0	27.96	25.63	30.29	
Foveolar hyperplasia	37 (10.0%)	1 (0.3%)	8 (2.2%)	18 (4.9%)	3 (8.1%)	1 (0.3%)	5 (1.4%)	26.29	23.80	29.41	*P* = .326 *Z* = −0.980
Intestinal metaplasia (stomach)	31 (8.4%)	0	10 (2.7%)	14 (3.8%)	4 (1.1%)	2 (0.5%)	1 (0.3%)	26.29	23.37	29.51	*P* = .859 *Z* = −0.177
Mucosal atrophy	14 (3.8%)	0	4 (1.1.%)	5 (1.4%)	3 (0.8 %)	1 (0.3%)	1 (0.3%)	27.31	24.30	32.03	*P* = .110 *Z* = −1.596
Barrett esophagus	20 (5.4%)	0	6 (1.6%)	10 (2.7%)	2 (0.5%)	0	0	26.36	24.56	28.08	*P* = .762 *Z* = −0.458

Missing BMI data: *N* = 27 (7.3%). Statistically significant values are indicated in bold.

BMI = body mass index, *H* = Kruskal–Wallis test, LA = Los Angeles, Me = median, Q1 = 1st quartile, Q3 = 3rd quartile, WHO = World Health Organization, *Z* = Mann–Whitney *U*-test.

Table 5 summarizes the diagnostic findings, but due to their rare occurrence, they were excluded in the statistical analyses but highlight the accuracy of the examinations. Notably, Table 5 also reflects observations in adult patients with visceral diseases, where comorbid celiac disease was not associated with undernutrition based on WHO criteria.

**Table 5:** Infrequent histopathological findings.

	Total	BMI class according to WHO						
		NA	Underweight	Standard	Overweight	Obese		
						Class I	Class II	Class III
HG dysplasia	1 (0.3%)	0	0	0	0	0	0	1 (0.3%)
LG dysplasia	2 (0.5%)	0	0	0	2 (0.5%)	0	0	2 (0.5%)
Polyps								
Elster cysts	13 (3.5%)	1 (0.3%)	0	4 (1.1%)	8	0	0	0
Hyperplastic	6 (1.6%)	1 (0.3%)	0	1 (0.3%)	4 (1.1%)	0	0	0
Inflammatory	1 (0.3%)	0	0	1 (0.3%)	0	0	0	0
Tubular adenoma	1 (0.3%)	0	0	0	0	0	0	1 (0.3%)
Xanthoma	1 (0.3%)	0	0	0	0	0	0	1 (0.3%)
Other lesions								
NET	1 (0.3%)	0	0	1 (0.3%)	0	0	0	0
Celiac disease	2 (0.5%)	0	0	2 (0.5%)	0	0	0	0

BMI = body mass index, HG = high-grade, LG = low-grade, NA = no data available, WHO = World Health Organization.

Table 6 shows the impact of sex on the incidence of panendoscopy-detected lesions. For analysis purposes, erosive esophagitis lesions were categorized into mild (LA grades A and B) and severe (LA grades C and D), with the latter meeting diagnostic criteria for GERD based on established guidelines. Although the evaluated groups did not reach statistical significance, the incidence of more severe forms of the disease was proportionally more frequent in men. Sex was associated with the incidence of lesions with regard to bile lakes (women: 79.3%, men: 20.7%; *P* = .004).

**Table 6:** The effect of sex on the prevalence of EGD pathologies.

	Prevalence		*P*-value
	Females (%)	Males (%)	
Barrett esophagus	12 (57.1%)	9 (42.9%)	.584
Intestinal metaplasia	21 (65.6%)	11 (34.4%)	.724
Bile lake	**46 (79.3%**)	**12 (20.7%**)	**.004**
Antral gastropathy (erosive)	26 (68.4%)	12 (31.6%)	.444
Antral gastropathy (erythematous/popular)	164 (60.7%)	99 (38.9%)	.554
Erosive esophagitis (LA grades A and B)	28 (59.6%)	19 (40.4%)	.630
Erosive esophagitis (LA grades C and D)	7 (53.9%)	6 (46.1%)	.499

*P*-value was determined using chi-squared test.

Significance level established at *P* = .05. Statistically significant values are indicated in bold.

Similarly, the prevalence of bile lakes was paid more attention. No statistically significant difference was observed in the BMI of women versus men with bile lakes. In addition, BMI had no overall on the incidence of bile lake. The correlation between BMI and intestinal metaplasia within the gastric mucosa was evaluated similarly, with no statistically significant correlation. Additionally, no statistically significant correlation was observed between the incidence of cholecystitis and Barrett esophagus or intestinal metaplasia incidence. Furthermore, no statistically significant relationship was observed between positive CLO screening results and the incidence of Barrett esophagus, intestinal metaplasia, and bile lakes (Tables 7 and 8).

**Table 7:** Influence of the presence of bile lake on the occurrence of intestinal metaplasia and Barrett esophagus.

	Bile lake		
	Prevalence	%	*P*
Barrett esophagus	4	8	.419
Intestinal metaplasia	7	14.0	.174

*P*-value was determined using chi-squared test.

**Table 8:** Effect of a positive CLO screening status on the prevalence of gastric intestinal metaplasia, Barrett esophagus, and bile lake.

	Positive CLO screening result		*P*-value
	Prevalence	%	
Barrett esophagus	7	6.0%	.736
Intestinal metaplasia	15	13.0%	.072

*P*-value was determined using chi-squared test.

No statistically significant correlations were observed with regard to the incidence of bile lakes. Nonetheless, the more frequent occurrence of intestinal metaplasia in patients with intestinal reflux is noteworthy. Although this test did not reach statistical significance, the occurrence of intestinal metaplasia being more common in patients with positive CLO screening results is noteworthy.

## 4. Discussion

For many decades, excess body weight is a risk factor for diaphragmatic hiatal hernia and the occurrence of esophagitis symptoms.^[31]^ A study showed that the prevalence of esophagitis was 18.92% of 1094 individuals deemed eligible for bariatric surgery. Although statistical analysis revealed no differences by sex or BMI, the group consisted of those who had already met the eligibility criteria for bariatric surgery.^[32]^ Despite the difference in the baseline characteristics of the study groups, our findings align closely, with the prevalence of this pathology estimated at 19.8%, and no differences being observed by sex. Notably, a correlation was found between the occurrence of this pathology and BMI, particularly in cases involving large hernia sizes. A University of California team made an analogous classification of hernia sizes following a study in 181 patients qualified for Roux-Y gastric bypass anastomosis. Within this cohort, the incidence of small hernias (>2 cm) was 32.6%, whereas large hernias were found in 4.4% of patients.^[33]^ Complementary insights into this issue were provided by Japanese researchers, who proved that increased gastric acid secretion plays a role in the pathogenesis of esophageal hiatal hernia in humans.^[34]^ However, we were unable to evaluate this phenomenon during routine gastroscopic examinations in this study.

In a study involving 5132 patients, Italian researchers found that statistically significant risk factors for erosive esophagitis were being overweight and presence of an esophageal hiatal hernia, although the sex was associated with nonerosive forms of reflux disease. Risk factors for Barrett esophagus included age >50 years, erosive forms of reflux disease, and male sex.^[35]^ In our study, erosive esophagitis (particularly LA grades C and D) was proportionally more frequent in men, but not statistically significant. Additionally, the incidence of Barrett esophagus was not affected by age, sex, and erosive esophagitis. Some studies showed that BMI does not affect the prevalence of GERD, hiatal hernia, or erosive esophagitis in the general population.^[36]^ Although the incidence of esophageal hiatal hernias seems to correlate with BMI, this correlation could not be confirmed in relation to the incidence of erosive esophagitis and Barrett esophagus.

Conversely, in our analyses, thyroid diseases were highlighted as a risk factor for Barrett esophagus. Oparin et al observed motility and secretory disorders in patients with GERD with concomitant autoimmune thyroiditis. The severity of GERD symptoms in these patients remained related to thyroid hormone levels, suggesting that changes in thyroid hormone metabolism are one of the factors involved in the mechanisms of GERD.^[37]^ This relationship presents an intriguing area for further investigation.

Regarding intestinal reflux, our study showed no statistically significant relationship between intestinal reflux and the occurrence of Barrett esophagus, most likely due to limited sample size. However, female sex and a history of thyroid disease were identified as significant risk factors for Barrett esophagus and intestinal reflux. Iwaya et al.^[38]^ involving 4021 patients demonstrated that endoscopic signs of biliary reflux were independent risk factors for Barrett esophagus.

The pH value, the presence of bile acids, and *H pylori* infection were most common factors influencing histological lesions in the gastric and esophageal mucosa. Some studies suggested the influence of smoking on the occurrence of metaplasia and atrophy in the gastric mucosa of patients infected with *H pylori*,^[39,40]^ which was consistent in our study group, although not statistically significant.

Shi focused on the possible relationship between bile reflux and *H pylori* infections. *H pylori* infection may increase gastrin secretion, decreasing peristalsis in the gastric antrum, thereby promoting bile reflux. Conversely, elevated pH values due to alkaline bile in the stomach, coupled with an impaired mucosal-bicarbonate barrier, may hinder the survival and colonization of *H pylori*. By contrast, a high bile acid concentration is a bactericidal factor.^[41]^ However, we could not confirm whether *H pylori* infection affects the incidence of biliary reflux.

Similarly, studies on young Greeks showed no high risk of *H pylori* infections in overweight and obese patients.^[42]^ Additionally, no increased incidence of *H pylori* infections was observed among patients awaiting bariatric surgery.^[43]^ However, Italian researchers showed that BMI and gastritis seem to be independent risk factors for unsuccessful *H pylori* eradication.^[44]^

Analyzing the frequency of the effects in chronic gastritis, Younes et al^[44]^ showed that intestinal metaplasia was less associated with *H pylori* infection and more strongly linked to reactive gastropathy, history of cholecystectomy, and higher BMI, indicating a stronger association with biliary reflux than with *H pylori*-related gastritis. In our evaluation, although not statistically significant, the incidence of intestinal metaplasia may be related to positive CLO results and bile reflux. Notably, obesity was independently associated with an increased incidence of atrophic gastritis and intestinal metaplasia in a group of Korean subjects.^[45]^ However, we could not confirm this finding for intestinal metaplasia in our cohort. In analyzing our results, a noticeable tendency (although not confirmed by statistical significance) emerged, indicating that atrophic gastritis was more prevalent with higher BMI. Exploring possible mechanisms for this relationship, attention is drawn to the role of ghrelin, which requires activation by the enzyme of ghrelin o-acyltransferase to perform its biological functions. Some studies have shown that eradicating *H pylori* infections increases ghrelin levels, improves appetite, and increases BMI.^[46,47]^ However, the higher prevalence of atrophic gastritis in those with higher BMI in our group does not support this hypothesis, indicating that the mechanism of this effect is rather multifactorial. Further analyses indicate higher ghrelin levels in the autoimmune form of atrophic gastritis and lower levels in those with advanced forms of atrophic inflammation,^[48]^ which could explain the correlation between atrophic gastritis and obesity in our study, and the lack of correlation between BMI and *H pylori* infection.

The prevalence of biliary reflux increases significantly after cholecystectomy, from 16.7% in the control group to 61.8% in postsurgery patients.^[49]^ In our study, this phenomenon was observed in 13.6% of patients, with a statistically significant correlation to cholecystectomy. Although gastritis is a common complication of cholecystectomy and may occur more frequently in patients with obesity and diabetes,^[50]^ we could not confirm this association. Lake et al.^[49]^ reported that cholecystectomy increases the risk of functional dyspepsia, biliary reflux, and abdominal pain but found no link between appendectomy on these symptoms. The occurrence of dyspepsia following cholecystectomy prompted patients to undergo testing. Sami-Bostan et al^[50]^ analyzed the diameter of the common bile duct (CBD) as a predictor of biliary reflux and noted a significant association between a CBD width of <7 mm and the occurrence of biliary reflux. However, no relationship was observed between CBD diameter and the frequency of inflammation, activity, atrophy, or *H pylori* infection. Interestingly, a correlation was found between CBD width and incidence of intestinal metaplasia. In contrast, our analysis revealed that the presence of a bile lake did not favor the occurrence of intestinal metaplasia in the gastric mucosa.

Given the low prevalence of upper GI malignancies (e.g., 3.6/100,000 for esophageal squamous cell carcinoma, 12.8/100,000 for gastric cancer), the absence of newly diagnosed GI tumors in our sample of 368 patients is consistent with epidemiological expectations.^[10,51]^

In conclusion, the incidence of upper gastrointestinal pathologies and their underlying pathomechanisms vary in patients undergoing bariatric procedures due to altered anatomical and biophysical relationships postoperatively. GERD represents a significant clinical concern in patients following laparoscopic sleeve gastrectomy, accounting for over 30% of patients during pH-metric examinations.^[52]^ Notably, successful weight reduction does not affect the complication rate in this patient group. Additionally, the absence of overt clinical symptoms does not exclude the presence of an endoscopic presentation of the disease.^[53]^ The occurrence of GERD in this patient population arises as a preexisting complication of obesity and as a de novo condition following surgery. Although GERD symptoms and erosive esophagitis have improved with pharmacological and surgical interventions, these treatments did not affect the incidence of Barrett esophagus.^[54]^

Biliary reflux is a significant concern following anastomosis gastric bypass surgery, with incidence ranging from 7.8% to 55%.^[55]^ Bernal highlighted 2 cases of malignant neoplasms in a 20-year period post-anastomosis gastric bypass, neither of which was correlated with biliary etiology. However, prospective studies involving preoperative endoscopy should be conducted preoperatively and at 5-year intervals to better understand the impact of bile exposure on the stomach and esophagus.^[54]^ The risk of carcinogenesis likely increases with prolonged bile exposure postoperatively.

The strengths of the presented study include the homogeneity of the group, characterized by a normal distribution of anthropometric characteristics. Including outpatients alone limited the influence of factors other than BMI, such as serious comorbidities. Due to the outpatient nature of the tests performed, chronic diseases had to be under control. Furthermore, a single physician examined all patients for a year, ensuring consistency in diagnostic standards and enabling the evaluation of seasonal variations in gastroenterological conditions.

Limitations of the study included the relatively small size of the study group, which poses a risk of error when extrapolating findings to larger populations, particularly given the rarity of the phenomena under investigation. Moreover, it also hindered achieving statistical significance in some assessments. Additionally, reliance on patient self-completion of the questionnaires led to incomplete interview data. Consequently, the effect of the drug treatment on the result of the endoscopic examination could not be evaluated, as the retrospective nature of the study did not allow for the collection of sufficiently reliable data for such analyses. Missing demographic and BMI data (*n* = 26) resulted from incomplete documentation during routine medical procedures, not from study design.

## 5. Conclusions

Our results showed that although BMI was strongly correlated with the presence of esophageal hiatal hernia, it was inversely associated with the incidence of intestinal reflux. Additionally, patients with mucosal atrophy and active inflammation exhibited higher median BMI than the study group, but this difference was not statistically significant. In contrast, comorbid thyroid diseases were associated with a higher incidence of bile reflux and Barrett esophagus, indicating a relationship warranting further investigation. Additionally, female sex was consistently associated with an incidence of biliary reflux into the stomach. Biliary reflux and *H pylori* infection contributed to a higher incidence of intestinal metaplasia in gastric mucosa and Barrett esophagus within the study group, although these associations were not statistically significant.

## Author contributions

**Conceptualization:** Przemysław Znamirowski.

**Data curation:** Przemysław Znamirowski.

**Formal analysis:** Przemysław Znamirowski.

**Funding acquisition:** Łukasz Nawacki.

**Investigation:** Przemysław Znamirowski.

**Methodology:** Przemysław Znamirowski.

**Resources:** Przemysław Znamirowski.

**Supervision:** Łukasz Nawacki, Stanisław Głuszek.

**Validation:** Łukasz Nawacki, Stanisław Głuszek.

**Writing – original draft:** Przemysław Znamirowski.

**Writing – review & editing:** Łukasz Nawacki.
